# Voxel‐based clustered imaging by multiparameter diffusion tensor images for predicting the grade and proliferative activity of meningioma

**DOI:** 10.1002/brb3.3201

**Published:** 2023-08-29

**Authors:** Yuki Takahashi, Naoya Oishi, Yukihiro Yamao, Takeharu Kunieda, Takayuki Kikuchi, Hidenao Fukuyama, Susumu Miyamoto, Yoshiki Arakawa

**Affiliations:** ^1^ Department of Neurosurgery Kyoto University Graduate School of Medicine Kyoto Japan; ^2^ Human Brain Research Center Kyoto University Graduate School of Medicine Kyoto Japan; ^3^ Department of Psychiatry Kyoto University Graduate School of Medicine Kyoto Japan; ^4^ Department of Neurosurgery Ehime University Graduate School of Medicine Toon Japan; ^5^ Yasu City Hospital Yasu Japan; ^6^ Kyoto University Kyoto Japan; ^7^ Stroke Support Center Kyoto University Hospital Kyoto Japan; ^8^ Momoya Disease Support Center Kyoto University Hospital Kyoto Japan

**Keywords:** diffusion tensor imaging, meningioma, support vector machine, voxel‐based clustering

## Abstract

**Introduction:**

Meningiomas are the most common primary central nervous system tumors. Predicting the grade and proliferative activity of meningiomas would influence therapeutic strategies. We aimed to apply the multiple parameters from preoperative diffusion tensor images for predicting meningioma grade and proliferative activity.

**Methods:**

Nineteen patients with low‐grade meningiomas and eight with high‐grade meningiomas were included. For the prediction of proliferative activity, the patients were divided into two groups: Ki‐67 monoclonal antibody labeling index (MIB‐1 LI) < 5% (lower MIB‐1 LI group; *n* = 18) and MIB‐1 LI ≥ 5% (higher MIB‐1 LI group; *n* = 9). Six features, diffusion‐weighted imaging, fractional anisotropy, mean, axial, and radial diffusivities, and raw T2 signal with no diffusion weighting, were extracted as multiple parameters from diffusion tensor imaging. The two‐level clustering approach for a self‐organizing map followed by the K‐means algorithm was applied to cluster a large number of input vectors with the six features. We also validated whether the diffusion tensor‐based clustered image (DTcI) was helpful for predicting preoperative meningioma grade or proliferative activity.

**Results:**

The sensitivity, specificity, accuracy, and area under the curve of receiver operating characteristic curves from the 16‐class DTcIs for differentiating high‐ and low‐grade meningiomas were 0.870, 0.901, 0.891, and 0.959, and those from the 10‐class DTcIs for differentiating higher and lower MIB‐1 LIs were 0.508, 0.770, 0.683, and 0.694, respectively. The log‐ratio values of class numbers 13, 14, 15, and 16 were significantly higher in high‐grade meningiomas than in low‐grade meningiomas (*p* < .001). With regard to MIB‐1 LIs, the log‐ratio values of class numbers 8, 9, and 10 were higher in meningiomas with higher MIB‐1 groups (*p* < .05).

**Conclusion:**

The multiple diffusion tensor imaging‐based parameters from the voxel‐based DTcIs can help differentiate between low‐ and high‐grade meningiomas and between lower and higher proliferative activities.

## Graphical abstract

To predict the grade and proliferative activity of meningiomas, we applied multiple parameters from preoperative diffusion tensor images to obtain a clustered image. The imaging‐based parameters can help differentiate between low‐ and high‐grade meningiomas and between lower and higher proliferative activities.

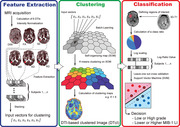

## 1 INTRODUCTION

Meningioma is the most common primary central nervous system tumor (Ostrom et al., 2013), histologically classified into three grades (I–III), according to the World Health Organization (WHO) classification (Louis et al., 2007). Grade II and III meningiomas have a greater risk of recurrence with increased mortality: the 5‐year survival rates are 78 and 44%, respectively (Durand et al., 2009). The most important prognostic question regarding meningioma concerns the prediction of recurrence after treatment. The histopathological grade of meningioma is currently the most useful morphological tool for predicting recurrence (Louis et al., 2007; Louis et al., 2016). The recurrence rates of grade II (atypical) and grade III (malignant) meningiomas at 5 years of follow‐up are about 40 and 50−80%, respectively (Santelli et al., 2010). Patients with malignant meningiomas have increased survival benefits if surgery is followed by fractionated external beam radiation therapy or stereotactic radiosurgery (Hanft et al., 2010). Therefore, preoperative characterization of meningiomas is significant in deciding therapy.

As another indicator of tumor growth, the Ki‐67 monoclonal antibody labeling index (MIB‐1 LI) has been reported to be useful (Matsuno et al., 1996). MIB‐1 LI has been extensively used in studies to determine the prognosis of several types of various tumors in central nervous system, including meningiomas, and an elevated MIB‐1 LI has been associated with an increased recurrence rate (Abry et al., 2010). Preoperative prediction by the value of MIB‐1 LI would lead us to optimal decision‐making about the resection area or enable us to decide, first of all, whether we should perform an operation or not.

Differentiation of cerebral tumor pathology currently relies on the interpretation of conventional structural magnetic resonance imaging (MRI). However, it is difficult to predict histological grade by conventional MRI (Demaerel et al., 1991). Diffusion‐weighted images (DWI) have been used to investigate primary brain neoplasms (Bulakbasi et al., 2004; Kono et al., 2001; Krabbe et al., 1997; Stadnik et al., 2001). Some studies have shown that atypical meningiomas show lower apparent diffusion coefficient (ADC) values compared with typical (benign) meningiomas (Filippi et al., 2001; Gupta et al., 2013; Hakyemez et al., 2006; Nagar et al., 2008), whereas other studies suggest that ADC values have no role in the preoperative grading of meningiomas (Santelli et al., 2010; Sanverdi et al., 2012). Diffusion tensor images (DTI) is a noninvasive technique that measures the random motion of water molecules across the brain due to thermal energy (Basser et al., 1994). It provides two common biomarkers of tissue microstructure: mean diffusivity (MD), which measures the magnitude of water molecule diffusion, and fractional anisotropy (FA), which measures directional coherence. Two other biomarkers provided by DTI are axial and radial diffusivities (AD and RD), which represent water diffusion parallel and perpendicular to the axonal fibers, respectively. From previous DTI studies (Bastin et al., 2002; De Belder et al., 2012; Jolapara et al., 2010; Sanverdi et al., 2012; Tang et al., 2014; Toh et al., 2008; Zikou et al., 2016), the parameters of DTI remain controversial for differentiating meningioma grade or proliferative parameters.

We recently reported a method of predicting glioma grade with a two‐level clustering approach, an unsupervised clustering method with a self‐organizing map (SOM) followed by K‐means (KM) (Inano et al., 2016, 2014). SOM is a well‐known type of neural network unsupervised learning that simplifies features and shows good visualization of results for data understanding and survey using component planes. In addition, features that have similar patterns can be identified by KM clustering of the results of SOM. This two‐level clustering approach has two important benefits in terms of noise reduction and computational cost (Inano et al., 2014). First, because the KM algorithm is very sensitive to outliers (Velmurugan & Santhanam, 2010), any outlier can adversely affect the accuracy of the clustering. When an SOM is applied prior to KM, the outliers can be filtered out, improving clustering accuracy. Second, the computational time of the two‐level clustering approach is considerably shorter than that of KM alone.

The purpose of our study was to apply the two‐level clustering method with multiple DTI‐based parameters for voxel‐based clustered images in meningiomas and to estimate whether the method is helpful for preoperative meningioma grading and differentiating high and low MIB‐1 LIs.

## 2 MATERIALS AND METHODS

### 2.1 Subjects

We retrospectively reviewed 114 patients aged 24−87 years who had histologically confirmed meningiomas, defined according to the WHO classification (Louis et al., 2007), and were treated between May 2014 and October 2016 at Kyoto University Hospital. Twenty‐nine patients underwent both DTI and three‐dimensional magnetization‐prepared rapid gradient‐echo (MP‐RAGE) preoperatively. We excluded one patient who was later found to have a solitary fibrous tumor by immunohistochemical staining and one patient because of appreciable motion artifacts on DTI. Consequently, 27 patients (aged 24−87 years, 13 men and 14 women) were enrolled in the study.

### 2.2 Histopathological diagnosis

The surgical samples were fixed with formalin, and paraffin‐embedded tissues were stained with hematoxylin and eosin (H&E) and MIB‐1. They were categorized into meningioma subtypes according to the WHO classification (David N. Louis et al., 2007). We classified grade I meningiomas as low‐grade (*n* = 19) and grade II and III meningiomas as high‐grade (*n* = 8) in this study. Among the low‐grade meningiomas, 12 were classified as meningothelial, three as transitional, two as fibrous, one as psammomatous, and one as angiomatous. Among the high‐grade meningiomas, six were classified as atypical and two as anaplastic. Nineteen patients reported no prior surgery, seven patients had a prior craniotomy, and one had undergone prior radiosurgery. There were no significant differences between the two groups in age (59.5 ± 11.9 years in the low‐grade group and 55.4 ± 19.5 years in the high‐grade group; *p* = .50) or sex ratio (eight males and 11 females in the low‐grade group and five males and three females in the high‐grade group; *p* = .33) (Table 1). MIB‐1 LIs were significantly higher in the high‐grade group (3.4 ± 4.5 in low‐grade and 14.1 ± 17.0 in high‐grade meningiomas; *p* = .015 by the Student's *t*‐test).

**TABLE 1:** Summary of data from patients with WHO grade I or II and III meningiomas.

Histopathology	*n*	WHO grade	Age (years)	Sex (M/F)	MIB‐1 LI (%)	Location
Total	27		58.3 ± 14.3	13/14	6.6 ± 10.8	
Grade I	19		59.5 ± 11.9	8/11	3.4 ± 4.5	
Meningothelial	12	I	58.0 ± 14.5		3.1 ± 4.5	Convexity, sphenoid ridge, parasagittal, intraventricular, planum sphenoidale, olfactory groove, petroclival
Transitional	3	I	60.0 ± 7.1		7.4 ± 5.8	Convexity, planum sphenoidale, petroclival
Fibrous	2	I	67.0 ± 0.0		2.0 ± 1.4	Parasagittal, intraventricular
Psammomatous	1	I	61		1.0	Parasagittal
Angiomatous	1	I	56		0.5	Convexity
Grades II, III	8		55.4 ± 19.5	5/3	14.1 ± 17.0	
Atypical	6	II	56.7 ± 21.0		5.5 ± 2.5	Convexity, parasagittal, petroclival
Anaplastic	2	III	51.5 ± 20.5		40.0 ± 14.4	Parasagittal, middle fossa

Age and MIB‐1 LI are given as means ± standard deviation.

MIB‐1 LI, Ki‐67 monoclonal antibody labeling index; WHO, World Health Organization.

Immunohistochemistry was performed with a fully automated multimodal slide‐staining system (Ventana Benchmark ULTRA) according to the manufacturer's protocols, using the following primary antibodies and dilutions: MIB‐1: monoclonal mouse Ki‐67 Clone MIB‐1/M7240, Dako, dilution 1:300. The largest representative tumor tissue section available was selected for immunohistochemistry. Diffuse glioma and normal tonsil tissue served as positive controls. Buffer without primary antibody was used for negative controls. The immunohistochemical stains were scored manually by neuropathologists independently from radiological findings. The anti‐Ki‐67 immunoreactive tissue section was scanned at low magnification to identify the area with the highest density of immunolabeled tumor cell nuclei (the “hot spot”). In these areas, tissue sections were examined at high‐power magnification (×400). The number of cells stained positively with MIB‐1 and the total number of tumor cells were counted in several representative fields containing nearly 1000 cells. Their ratio was indicated as MIB‐1 LI (%). In the areas of heterogeneous distribution of MIB‐1 immunopositive cells, the area containing the largest number of MIB‐1 immunostained cells was regarded as the area representing the proliferative activity of the tumor. For the prediction of meningioma recurrence, the patients were divided into two groups: MIB‐1 LI < 5% (lower MIB‐1 LI group; *n* = 18) and MIB‐1 LI ≥ 5% (higher MIB‐1 LI group; *n* = 9) (Table 2). The MIB‐1 LI cutoff value was adopted for the detection of meningiomas with more aggressive features (Abramovich & Prayson, 1998; Abry et al., 2010; Ohta et al., 1994). The lower MIB‐1 LI group consisted of 16 low‐grade and two high‐grade meningiomas, and the higher MIB‐1 LI group consisted of three low‐grade and six high‐grade meningiomas. The distribution of WHO grades was significantly different between the two groups (*p* = .002, chi‐squared test). There were no significant differences between the two groups in age (59.8 ± 14.0 years in the lower MIB‐1 group and 55.3 ± 15.3 years in the higher MIB‐1 group; *p* =.45) or sex ratio (nine males and nine females in the lower MIB‐1 group and four males and five females in the higher MIB‐1 group; *p* = .78) (Table 2).

**TABLE 2:** Summary of data from patients with MIB‐1 LI <5% and ≥5% meningiomas.

Histopathology	*n*	WHO grade	Age (years)	Sex (M/F)	MIB‐1 LI (%)	Location
Total	27		58.3 ± 14.3	13/14	6.6 ± 10.8	
MIB‐1 LI < 5%	18		59.8 ± 14.0	9/9	1.9 ± 1.2	
Meningothelial	11	I	57.9 ± 15.2	6/5	1.8 ± 1.2	Convexity, sphenoid ridge, parasagittal, intraventricular, planum sphenoidale, petroclival
Transitional	1	I	68	0/1	3.0	Planum sphenoidale
Fibrous	2	I	67.0 ± 0.0	0/2	2.0 ± 1.4	Parasagittal, intraventricular
Psammomatous	1	I	61	0/1	1.0	Parasagittal
Angiomatous	1	I	56	1/0	0.5	Convexity
Atypical	2	II	60.0 ± 28.3	2/0	3.0 ± 1.4	Parasagittal, petroclival
MIB‐1 LI ≥5%	9		55.3 ± 15.3	4/5	15.9 ± 15.1	
Meningothelial	1	I	62	0/1	16.9	Olfactory groove
Transitional	2	I	56.5 ± 3.5	1/0	9.7 ± 6.2	Convexity, petroclival
Atypical	4	II	55.0 ± 21.4	3/1	6.7 ± 2.0	Convexity, parasagittal
Anaplastic	2	III	51.5 ± 20.5	0/2	40.0 ± 14.1	Parasagittal, middle fossa

Age and MIB‐1 LI are given as means ± standard deviation.

MIB‐1 LI, Ki‐67 monoclonal antibody labeling index; WHO, World Health Organization.

### 2.3 MRI data acquisition and preprocessing

MRI data were acquired on a 3 Tesla Trio Tim (Siemens) equipped with a 32‐channel phased‐array head coil. DWI in an axial orientation used the following parameters: repetition time = 4200 ms, echo time = 104 ms, flip angle = 90°, field of view = 192 × 192 mm, slices = 78, and voxel size = 1.7 × 1.7 × 1.7 mm. To resolve the geometrical distortions, we acquired two images for each diffusion gradient: one with anterior–posterior and one with posterior–anterior k‐space in the phase‐encode direction. DWI was isotropically distributed along 40 directions using a *b*‐value of 1000 s/mm^2^. Eight volumes with no diffusion weighting (*b* = 0 s/mm^2^) were also acquired at points throughout acquisition. MP‐RAGE using the following parameters was used to acquire three‐dimensional T1‐weighted anatomical images: repetition time = 1900 ms, echo time = 2.58 ms, flip angle = 9°, field of view = 230 × 230 mm, slices = 192, and voxel size = 0.9 × 0.9 × 0.9 mm. A dual‐gradient field map in an axial orientation was also obtained using the following parameters: repetition time = 1100 ms, echo time = 35 ms, flip angle = 60°, field of view = 192 × 192 mm, slices = 46, and voxel size = 3 × 3 × 3 mm. Regions of interest (ROIs) for analysis were manually traced in the DTI space according to abnormalities on MP‐RAGE and well‐circumscribed gadolinium‐enhanced T1‐weighted images, obtained on another 3T machine (Skyra; Siemens) using the following parameters: repetition time = 1900 ms, echo time = 2.58 ms, flip angle = 9°, field of view = 229 × 229 mm, slices = 58, and voxel size = 0.9 × 0.9 × 1.0 mm.

The DTI data were analyzed by FSL [FMRIB Software Library v5.0.9, http://www.fmrib.ox.ac.uk/fsl (Smith et al., 2004)]. The data were corrected for eddy currents and head motion using affine registration to the first *b* = 0 reference volume. The data were also corrected for geometric distortions occurring in an echo planar image (Jezzard & Balaban, 1995) by TOPUP, which is a part of the FSL tool for correction of distortions (Andersson et al., 2003). Six features, that is, DWI, FA, MD, AD, RD, and raw T2 signal with no diffusion weighting (S0), were extracted using the FMRIB diffusion toolbox (FDT) program (Smith et al., 2004), as reported previously (Inano et al., 2016, 2014).

### 2.4 Feature extraction for clustering

**FIGURE 1:**
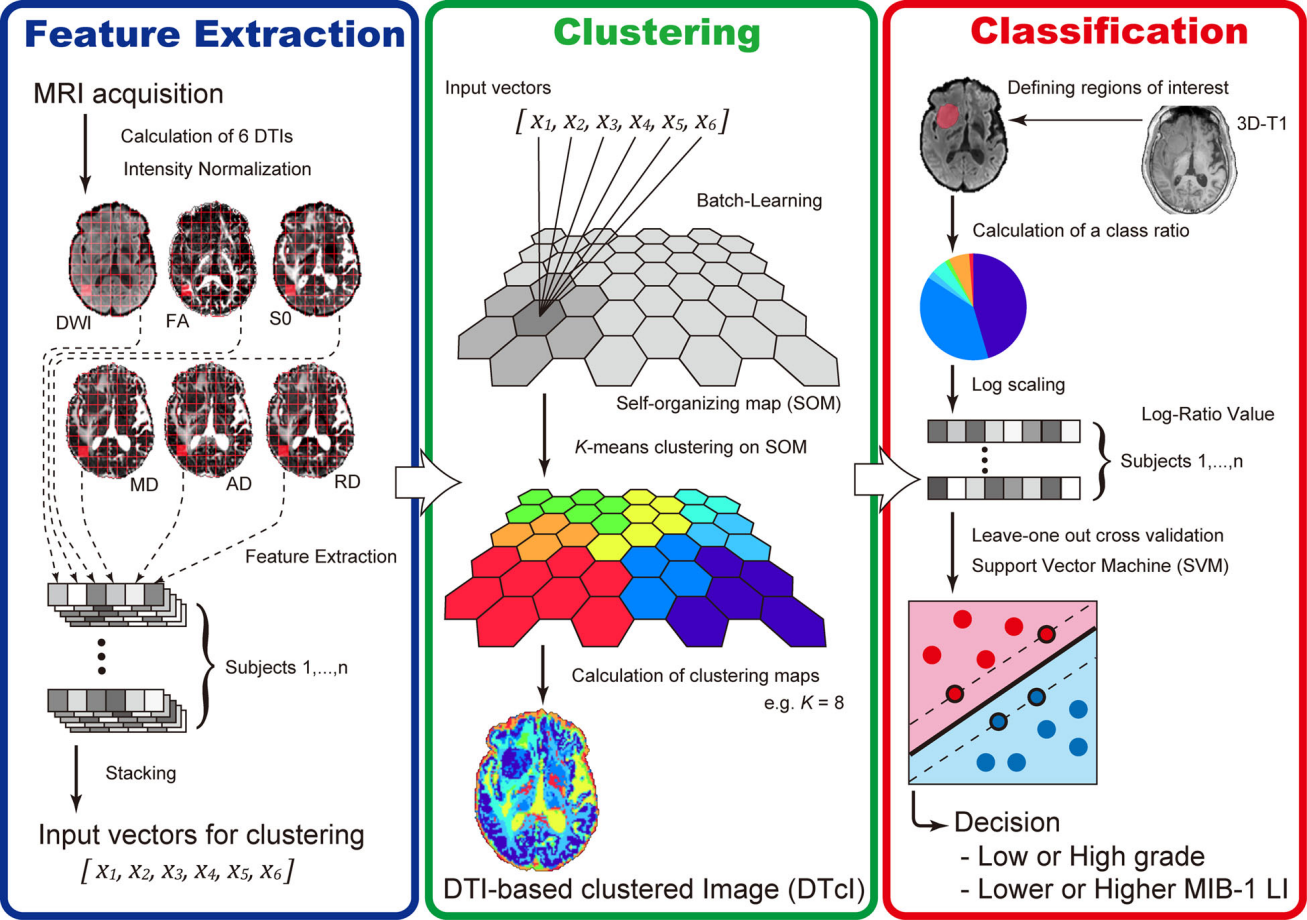
Simplified graphical overview of the processing pipeline.

The details of the clustering methods were described previously (Inano et al., 2014). The summary of the processing pipeline is as follows (Figure 1).

1. Feature extraction from DTI (the number of extracted features was 9564 ± 4260 for each subject).
2. Clustering using SOM followed by KM.
3. Visualization of whole‐brain images by diffusion tensor‐based clustering images (DTcIs).
4. Classification using DTcIs by the support vector machine (SVM).

### 2.5 Definition of ROI

Meningiomas generally show clear and smooth boundaries, but some of them infiltrate surrounding brain matter and have unclear boundaries and discontinuities. ROIs were manually traced in the DTI space according to abnormalities on MP‐RAGE, with reference to well‐circumscribed gadolinium‐enhanced T1‐weighted images, without any knowledge of the clinical or pathological data. It should be noted that ROIs were defined on the basis of abnormal signal intensities in MP‐RAGE and gadolinium‐enhanced T1‐weighted images, excluding perifocal edema and surrounding cerebrospinal fluid but including cystic parts of the tumors. Finally, the number of voxels in each ROI ranged from 100 to 17,463. We only used these ROIs for feature extraction for the SVM.

### 2.6 Statistical analysis

As reported previously (Inano et al., 2014), to determine whether the classification performances were significantly different according to the number of *K* in the KM++ method (*K* = 4, 6, 8, 10, 12, 16, 20), we repeated the leave‐one‐out cross‐validation (LOOCV) strategy 100 times. The area under the curve (AUC) in different *K* were then analyzed by one‐way ANOVA followed by Tukey's multiple comparison tests. *p* < .05 was considered significant.

To evaluate the behavior of the classifier in the *K* class that showed the best classification performance, we used the “pROC library” for R to generate receiver operating characteristic curves with 95% confidence intervals (CIs) (Robin et al., 2011). Wilcoxon–Mann–Whitney tests with exact *p* values and CIs calculated by a permutation test were used to compare the log‐ratio values of each class in the *K* class.

The ratios of normalized intensities on the six DTI of each class in the *K* class that showed the best classification performance were analyzed with the bootstrapped 95% CIs. The statistical software package R version 3.0.2 (The R Foundation for Statistical Computing, http://www.r‐project.org/) was used to perform all statistical analyses.

## 3 RESULTS

### 3.1 Unsupervised clustering

**FIGURE 2:**
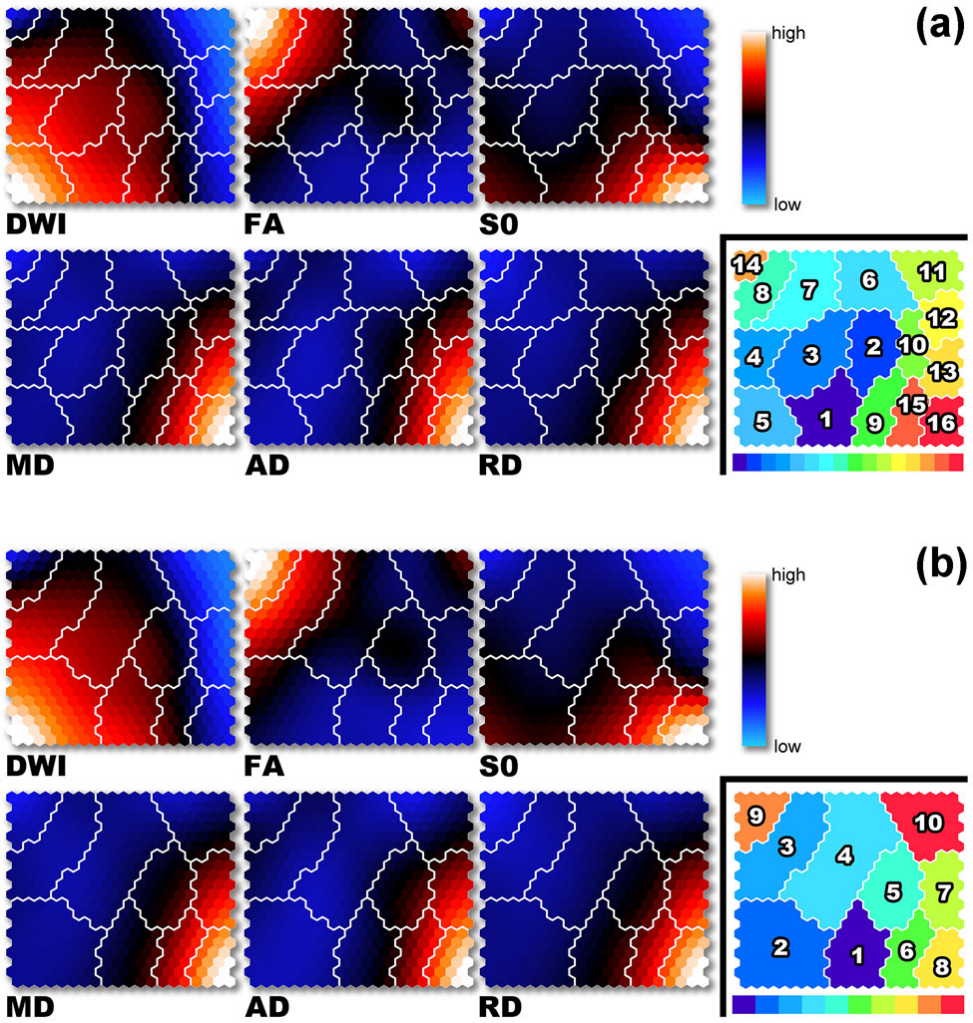
Visualization of six DTI‐based variables on component planes with 20 × 20 SOM. Each node (protocluster) is colorized from blue to red according to the intensities in each diffusion tensor image. The white lines between nodes denote interclass borderlines obtained by KM++ with *K* = 16 (a) and *K* = 10 (b) on SOM. SOM component planes can help to interpret detailed intensity profiles or patterns in each diffusion tensor image. Each class number corresponds to the intensity on DTI‐based clustered images. DTI, diffusion tensor MRI; SOM, self‐organizing map; DWI, diffusion‐weighted imaging; FA, fractional anisotropy; S0, raw T2 signal without diffusion weighting; MD, mean diffusivity; AD, axial diffusivity; RD, radial diffusivity.

Figure 2 illustrates the component planes in six DTI‐based variables by SOM analysis. Visual inspection of the SOM patterns demonstrated that the component planes of DWI and FA were obviously different from the others. Although the general patterns of the S0, MD, AD, and RD component planes seemed similar, the details differed among them. In the case of *K* = 16 (Figure 2a), for example, the FA values in class number 14, the DWI values in class number 5, and the S0, MD, AD, and RD values in class number 16 were the highest among all classes. In the case of *K* = 10 (Figure 2b), the FA values in class number 9, the DWI values in class number 2, and the S0, MD, AD, and RD values in class number 8 were the highest among all classes. SOM also showed that the DWI components of class numbers 8, 6, and 15 (*K* = 16) and class numbers 3, 4, 5, 6, and 9 (*K* = 10) varied from low to high values, and that the FA components of class numbers 3, 6, and 11 (*K* = 16) and class numbers 2, 4, and 10 (*K* = 10) varied from low to high values. Class numbers 1, 2, 4, and 5 in the S0 component plane were higher than those in the MD, AD, and RD component planes (*K* = 16). Class numbers 10, 12, and 13 in the MD, AD, and RD component planes were higher than those in the S0 component plane (*K* = 16).

**FIGURE 3:**
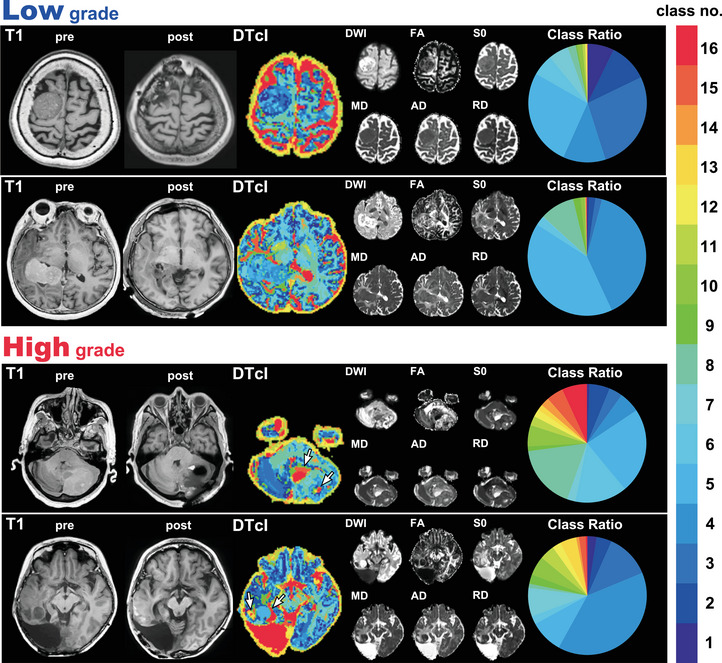
Representative cases of low‐ and high‐grade meningiomas, including the 16‐class DTcIs that showed the highest classification performance. The preoperative and postoperative T1‐weighted images, DTcIs, six diffusion tensor images, and the ratios in each class number are shown for each patient. The arrows show the areas of warm‐colored classes, which indicate WHO high grades. The colors on the DTcIs and circular charts correspond to the class numbers shown in the color bar. DWI, diffusion‐weighted imaging; FA, fractional anisotropy; S0, raw T2 signal without diffusion weighting; MD, mean diffusivity; AD, axial diffusivity; RD, radial diffusivity; DTcI, diffusion tensor‐based clustering image; WHO, World Health Organization.

Representative cases of low‐grade and high‐grade meningiomas are shown in Figure 3. Almost all the boundaries of low‐grade meningiomas could be clearly recognized, but it was much more difficult to recognize the boundaries of high‐grade meningiomas. In high‐grade meningiomas, DTcIs revealed warm‐colored classes, such as class numbers 13, 14, 15, and 16, mainly in their tumor cysts or probably in the necrotic region. Thus, the clear differentiation between low‐grade and high‐grade meningiomas on DTcIs could be visually recognized.

**FIGURE 4:**
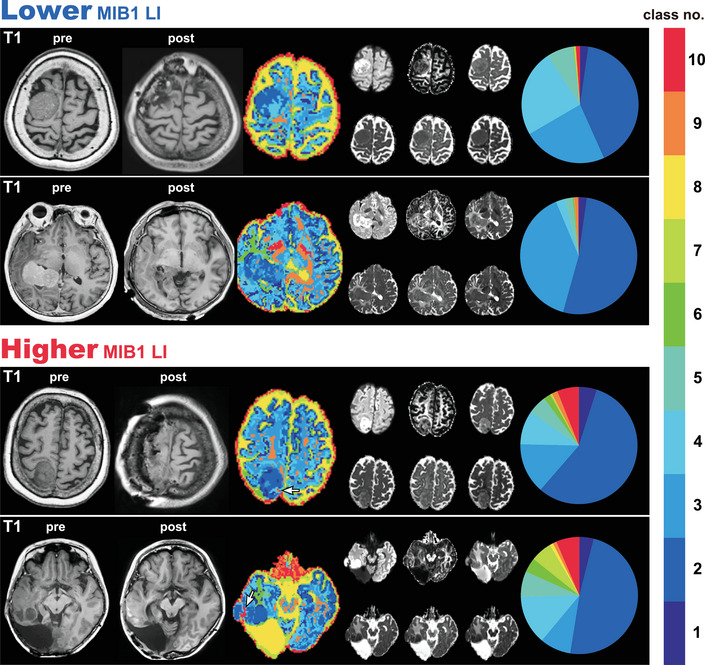
Representative cases of meningiomas with lower and higher MIB‐1 LIs, including the 10‐class DTcIs that showed the highest classification performance. The preoperative and postoperative T1‐weighted images, DTcIs, six diffusion tensor images, and the ratios in each class number are shown for each patient. The arrows show the areas of warm‐colored classes, which indicate the areas with higher MIB‐1 LI. The colors on the DTcIs and circular charts correspond to the class numbers, which are shown in the color bar. MIB‐1 LI, Ki‐67 monoclonal antibody labeling index; DWI, diffusion‐weighted imaging; FA, fractional anisotropy; S0, raw T2 signal without diffusion weighting; MD, mean diffusivity; AD, axial diffusivity; RD, radial diffusivity; DTcI, diffusion tensor‐based clustering image.

**FIGURE 5:**
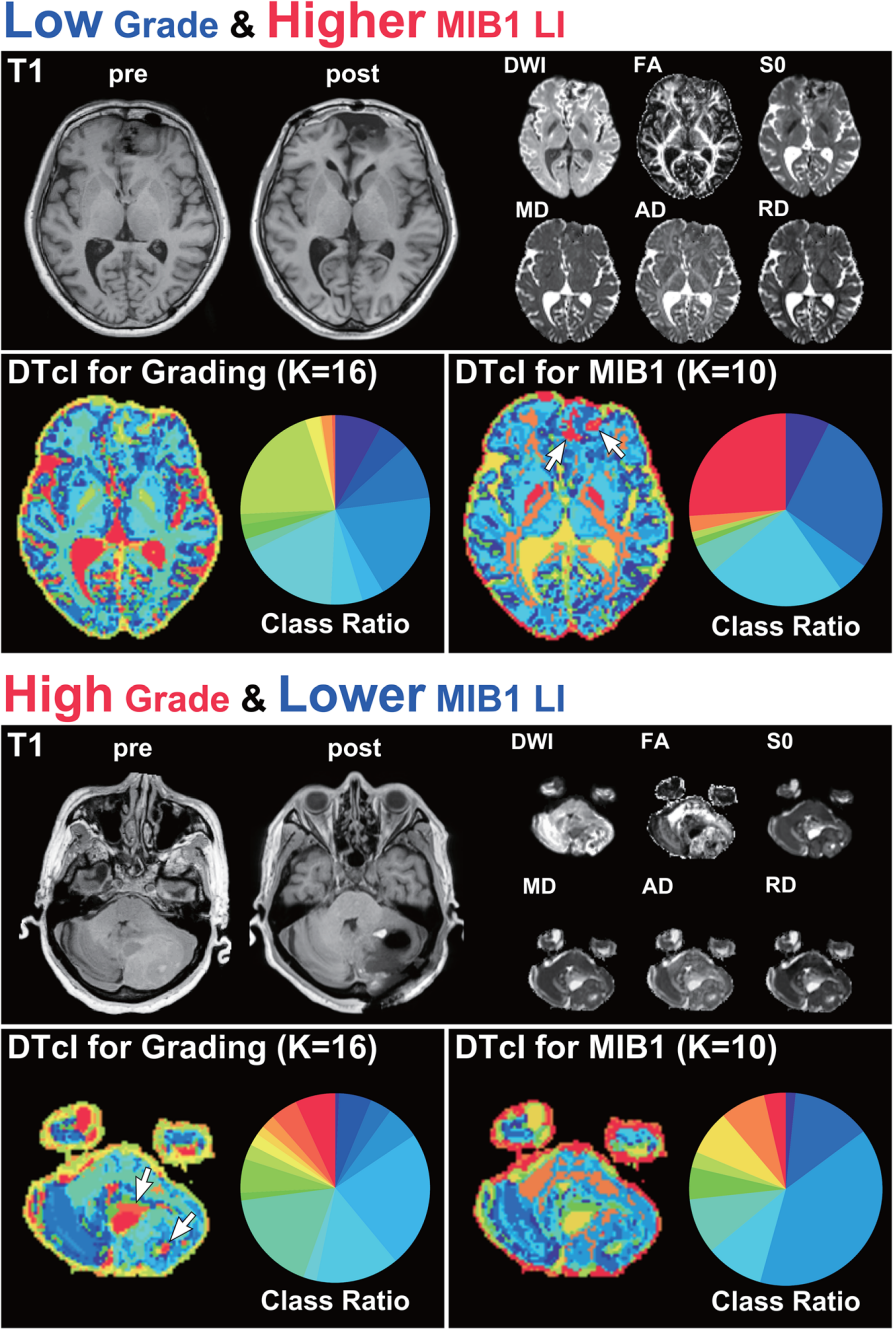
Representative cases of 16‐ and 10‐class DTcIs, which respectively showed the highest performance for classification between low‐ and high‐grade meningiomas and lower and higher MIB‐1 LIs, of a low‐grade meningioma with a higher MIB‐1 LI (upper) and a high‐grade meningioma with a lower MIB‐1 LI (lower). The upper and lower arrows show the areas of warm‐colored classes, which indicate a meningioma with higher MIB‐1 LI and a high‐grade meningioma, respectively. MIB‐1 LI, Ki‐67 monoclonal antibody labeling index; DTcI, diffusion tensor‐based clustering image; DWI, diffusion‐weighted imaging; FA, fractional anisotropy; S0, raw T2 signal without diffusion weighting; MD, mean diffusivity; AD, axial diffusivity; RD, radial diffusivity.

In general, DTcIs could also distinguish the lesions of meningiomas with higher MIB‐1 LIs (Figure 4). The area with warm‐colored classes (class numbers 8, 9, and 10) seemed to roughly correspond with the area shown as warm colors in the WHO grading (class numbers 13, 14, 15, and 16) (Figure 4). Although a warm‐colored pattern in a low‐grade meningioma with a higher MIB‐1 LI seemed similar in the images of *K* = 16 (WHO grading) and *K* = 10 (MIB‐1 LI), there were some differences in detail (Figure 5). The area with warm color in *K* = 10 (higher MIB‐1 LI) was also warm colored in *K* = 16 (WHO grading); however, the colors were mostly green or yellow, which is not indicative of a high WHO grade. The result was similar in a patient with high‐grade meningioma with a lower MIB‐1 LI. The area with hot color in *K* = 16, which was assumed to be high‐grade in the WHO grading, was green or yellow in *K* = 10, which did not indicate higher MIB‐1 LIs.

### 3.2 SVM classification using DTcI

**FIGURE 6:**
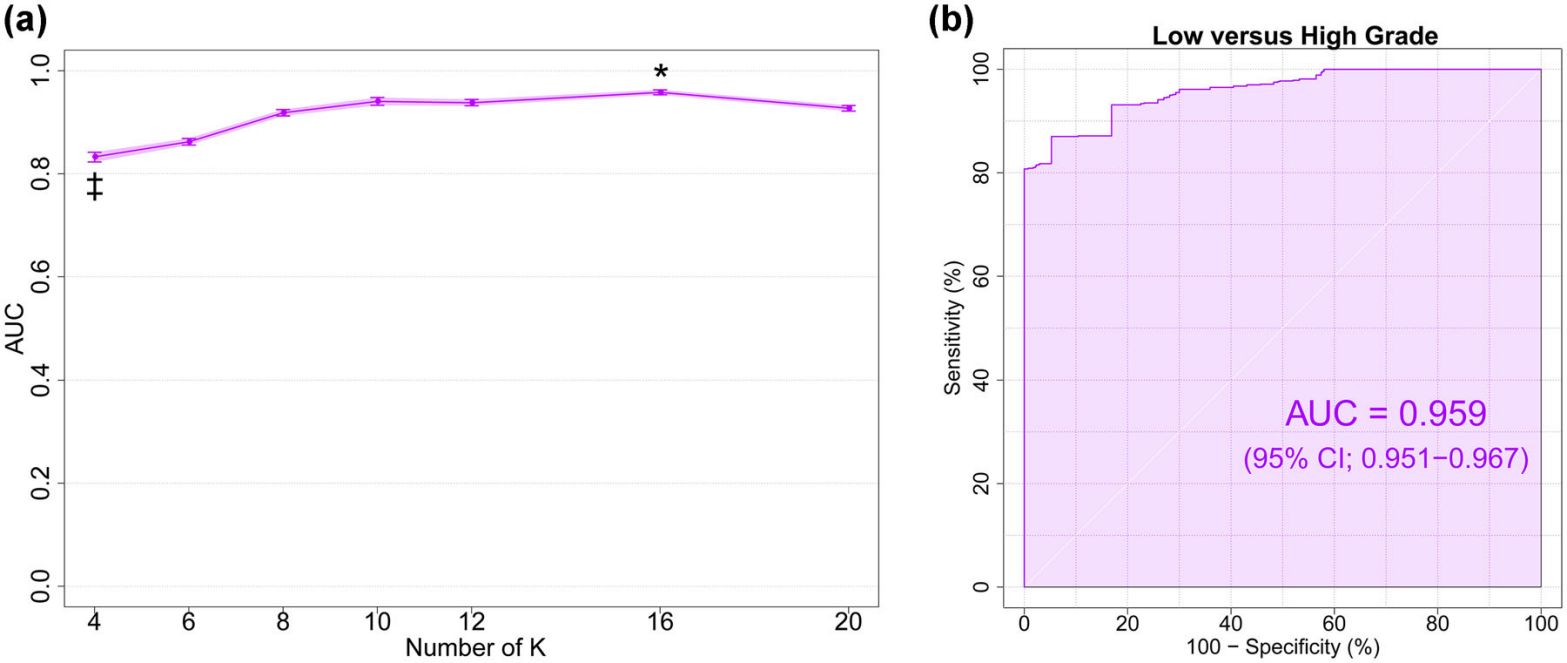
(a) Plots of AUC versus the number of K in the KM++ method for differentiating meningioma grades. Values are means and error bars, and light purple shading represents 95% CIs. One‐way ANOVA followed by Tukey's multiple comparison tests. The 16‐class DTcIs significantly showed the highest AUC (0.959; 95% CI = 0.951−0.967). (b) ROC curves (dark purple line), with AUC and 95% CIs shown in purple shading surrounding the dark purple line, for differentiating high‐grade from low‐grade meningiomas using the 16‐class DTcIs. AUC, area under the curve; CI, confidence interval; ANOVA, analysis of variance; DTcI, diffusion tensor‐based clustered image; ROC, receiver operating characteristic.

The performance of LOOCV using DTcI and SVM is shown in Figure 6a. The differences in AUC among the classes were significant [*F*(6, 693) = 181.11, *p* < 10^−138^, $\eta_{p}^{2}\;$= 0.61]. Tukey's post hoc tests showed that the AUC was significantly higher for the 16‐class DTcIs than for the others (*p* < .001). The tests showed that the AUCs were significantly higher for the 8‐, 10‐, 12‐, and 20‐class DTcIs than for the 4‐ and 6‐class DTcIs (*p* < .001). The tests also showed that the AUC was significantly lower for the 4‐class DTcIs than for the others (*p* < .001). The AUC of the 16‐class DTcIs was the highest among classes (0.959; 95% CI = 0.951−0.967) (Figure 6b). The sensitivity, specificity, and accuracy of the 16‐class DTcIs were 0.870 (95% CI = 0.865−0.873), 0.901 (95% CI = 0.897−0.904), and 0.891 (95% CI = 0.889−0.894), respectively. In contrast, the AUC of the 4‐class DTcIs was the lowest (0.833; 95% CI = 0.814−0.853).

**FIGURE 7:**
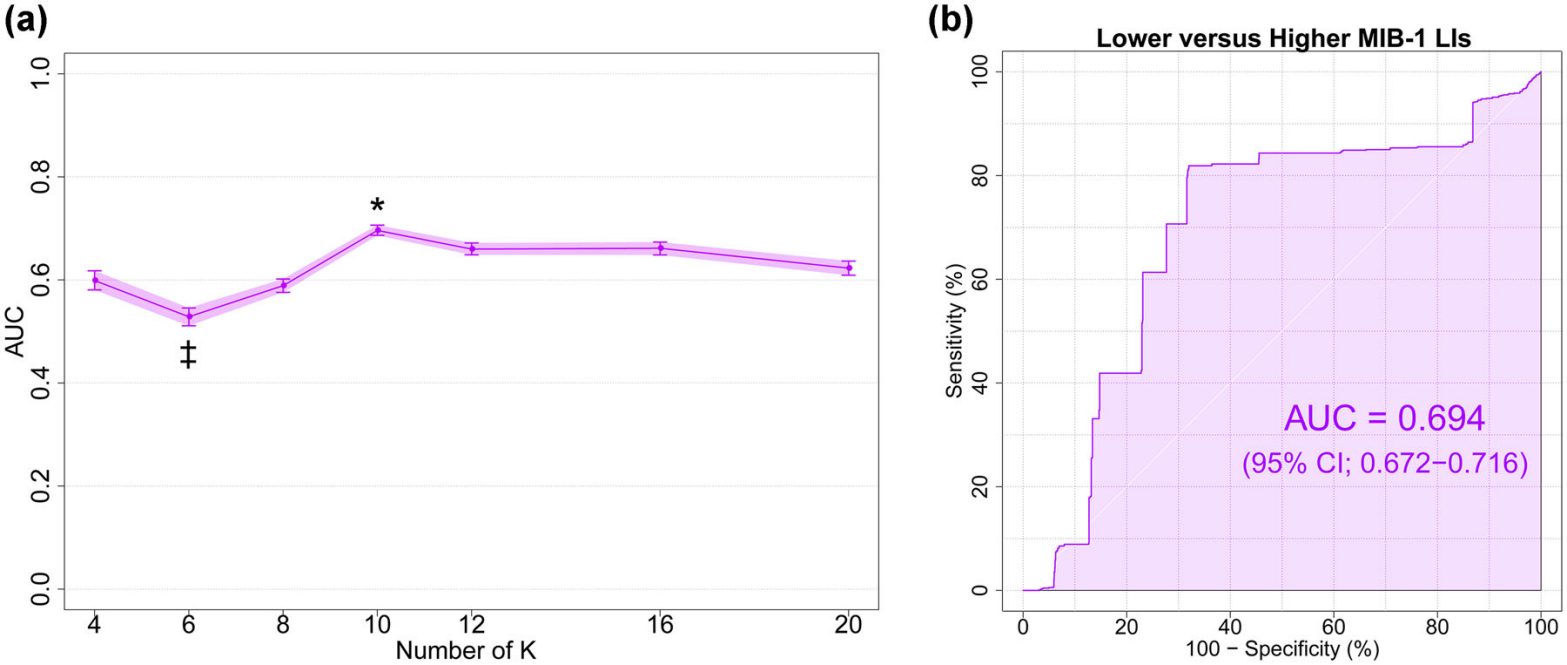
(a) Plots of AUC versus the number of K in the KM++ method for MIB‐1 LI grouping. Values are means and error bars, and light purple shading represents 95% CIs. One‐way ANOVA followed by Tukey's multiple comparison tests. The 10‐class DTcIs significantly showed the highest AUC (0.694; 95% CI, 0.672−0.716). (b) ROC curves (dark purple line), with AUC and 95% CIs shown in purple shading surrounding the dark purple line, for differentiating meningiomas with an MIB‐1 LI threshold of 5% using the 10‐class DTcIs. AUC, area under the curve; MIB‐1 LI, Ki‐67 monoclonal antibody labeling index; CI, confidence interval; ANOVA, analysis of variance; DTcI, diffusion tensor‐based clustering image; ROC, receiver operating characteristic.

We separated the patients into two MIB‐1 LI groups with a threshold of 5.0% (Abramovich & Prayson, 1998, 1999; Abry et al., 2010). The results are shown in Figure 7a. The differences in AUCs among the classes were significant [*F*(6, 693) = 60.349, *p* < 10^−60^, $\eta_{p}^{2}$ = 0.34]. Tukey's post hoc tests showed that AUC was significantly higher for the 10‐class DTcIs than for the others (*p* < .001). The tests also showed that the AUC was significantly lower for the 6‐class DTcIs than for the others (*p* < .001). The AUC of the 10‐class DTcIs was the highest among classes (0.694; 95% CI = 0.672−0.716) (Figure 7b). The sensitivity, specificity, and accuracy of the 10‐class DTcIs were 0.508 (95% CI = 0.493−0.523), 0.770 (95% CI = 0.766−0.774), and 0.683 (95% CI = 0.677−0.688), respectively.

### 3.3 Differences in log‐ratio values

**FIGURE 8:**
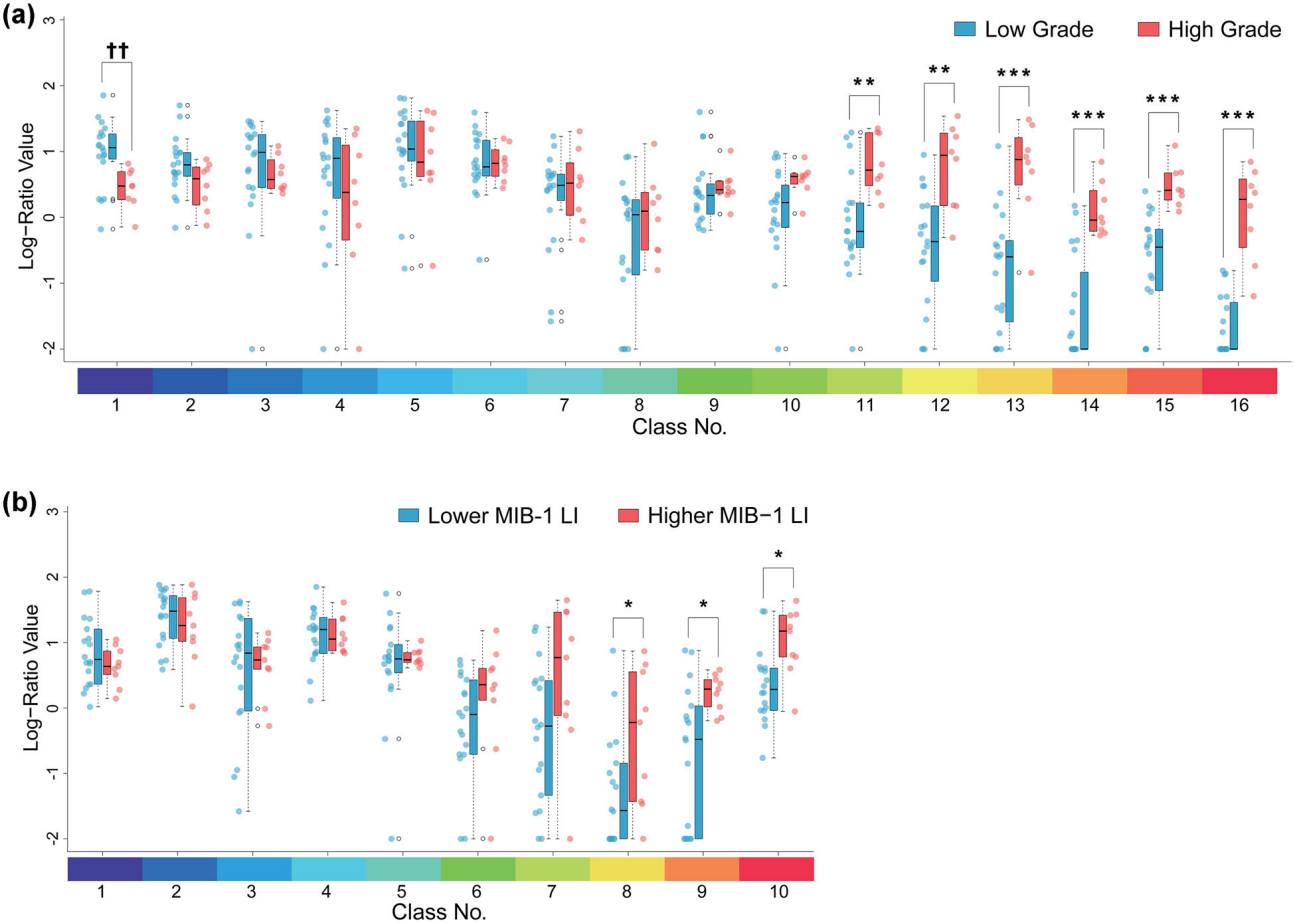
Strip chart and box plots showing the median, interquartile range, inner fence, and outliers (open circles) for log‐ratio values of each class by 16‐class DTcIs in patients with low‐grade (blue) and high‐grade (red) meningiomas (a), and by 10‐class DTcIs in patients with MIB‐1 LIs <5% (blue) and ≥5% (red) (b). *p < .05, **p < .005, ***p < .001, ††*p* < .005 by the exact Wilcoxon–Mann–Whitney rank sum test. DTcI, diffusion tensor‐based clustered image; MIB‐1 LI, Ki‐67 monoclonal antibody labeling index.

The log‐ratio values of each class of the 16‐class DTcIs that had the highest classification performance were compared between low‐grade and high‐grade meningiomas (Figure 8a). The values of class numbers 13, 14, 15, and 16 were significantly higher in high‐grade meningiomas than in low‐grade meningiomas (*p* < .001, *r* = 0.61; *p* < .001, *r* = 0.69; *p* < .001, *r* = 0.73; *p* < .001, *r* = 0.77, respectively). The values of class numbers 11 and 12 were also higher in high‐grade meningiomas (*p* < .005, *r* = 0.55; *p* < .005, *r* = 0.58, respectively). However, the value of class number 1 was higher in low‐grade meningiomas (*p* < .005, *r* = 0.50) than in high‐grade meningiomas.

With regard to MIB‐1 LIs, the log‐ratio values of each class of the 10‐class DTcIs were compared with the threshold of 5% (Figure 8b). The values of class numbers 8, 9, and 10 were higher in meningiomas with higher MIB‐1 LIs (*p* < .05, *r* = 0.38; *p* < .05, *r* = 0.45; *p* < .05, *r* = 0.46, respectively).

### 3.4 Ratio of DTI‐based parameters

**FIGURE 9:**
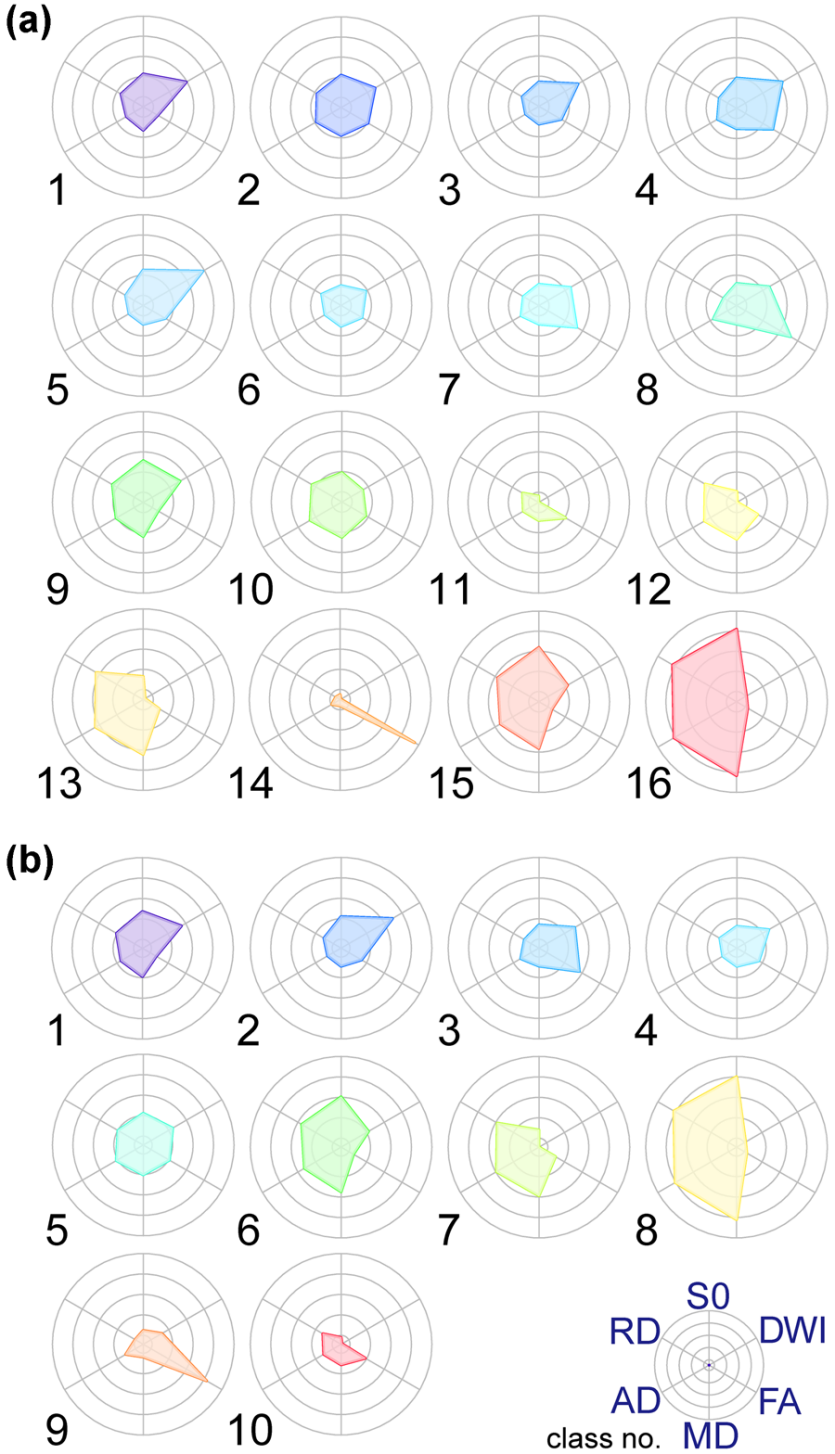
Radar charts of six DTI‐based variables in each class by 16‐class DTcIs (a) and 10‐class DTcIs (b), whose AUCs were the highest for WHO grading and MIB‐1 LI grouping, respectively. DTI, diffusion tensor MRI; DTcI, diffusion tensor‐based clustering image; AUC, area under the curve; WHO, World Health Organization; MIB‐1 LI, Ki‐67 monoclonal antibody labeling index; DWI, diffusion‐weighted imaging; FA, fractional anisotropy; S0, raw T2 signal without diffusion weighting; MD, mean diffusivity; AD, axial diffusivity; RD, radial diffusivity.

In the comparison of low‐grade and high‐grade meningiomas, the ratios of the normalized intensities of the six DTIs for each class number in the 16‐class DTcIs that showed the highest classification performance are shown in Figure 9a. The ratios of class numbers 13, 14, 15, and 16 were significantly higher in high‐grade meningiomas than in low‐grade meningiomas. The chart patterns of class number 16 comprised high S0, MD, AD, and RD values, whereas DWI and FA were quite low. Class number 15 also showed low FA values; however, the variables of class number 14 included prominently high FA values. The quite low values of DWI of class number 13 stood out against high AD, MD, and RD values. All these characteristics were composed of multiple parameters and were difficult to explain by brief visual inspection and by a distinct trend of a single parameter.

With regard to MIB‐1 LI, the ratios of class numbers 8, 9, and 10 tended to be higher in meningiomas with higher MIB‐1 LIs (Figure 9b). The chart patterns of class number 10 comprised low DWI, FA, S0, MD, AD, and RD values, and class number 9 had quite high FA values. The patterns of class numbers 6, 8, and 10 were very similar to those of class numbers 15, 16, and 11 in the 16‐class DTcIs, respectively. However, the pattern of class number 9 was different from that of class number 14 in the 16‐class DTcIs, whereas its FA values were the highest.

## 4 DISCUSSION

We investigated a two‐level clustering approach using SOM followed by KM to identify the WHO grade and higher MIB‐1 LI values of meningiomas. DTcIs enabled us to predict the meningioma grades even though they were calculated from preoperative images. Next, we assessed the validity of DTcIs for meningioma grading in a supervised manner using SVM. The 16‐class DTcIs had the highest classification performance for predicting meningioma grade, and the 10‐class DTcIs had the highest classification performance for MIB‐1 LI grouping. The classifier in the 16‐class DTcIs showed that the ratios of classes 13−16 were significantly higher in high‐grade meningiomas and those of class 1 were significantly higher in low‐grade meningiomas. These results indicate that our clustering method with six DTI‐derived parameters can be useful for predicting meningioma grade visually. The comparatively homogeneous structures of meningiomas would lead to good results, although the number of subjects is less than that in our previous study of gliomas (Inano et al., 2014). With regard to MIB‐1 LIs, classes 8, 9, and 10 indicate a higher MIB‐1 LI group. The sensitivity, specificity, accuracy, and AUC of the 10‐class DTcIs for differentiating between lower and higher MIB‐1 LIs were not as high as those of 16‐class DTcIs for differentiating between low‐ and high‐grade meningiomas; however, the 10‐class DTcIs would be possible to predict areas with higher MIB‐1 LIs.

Class numbers 13−16 with hotter colors in DTcIs, which indicate high‐grade meningiomas, seem to be found in tumor cysts or necrosis. This is consistent with previous reports of conventional studies of the use of MRI for meningioma grading (Hsu et al., 2010; Radeesri & Lekhavat, 2023). Conventional MRI can provide information for the diagnose of meningiomas due to its high resolution, but sometimes fails to provide a differentiation of meningiomas’ grade due to the overlap of imaging features (She et al., 2023). Thus, radiomics including this two‐level clustering approach can extract and analyze numerous high‐level, quantitative imaging features to differentiate the grade of meningiomas. Although the area with hot color in 16‐class DTcIs of the WHO grading basically overlapped with the hot area in 10‐class DTcIs about MIB‐1 LIs, the hot areas in 10‐ or 16‐class DTcIs did not always correspond with each other (Figures 3 and 4). Furthermore, some patients with high‐grade meningiomas could have lower MIB‐1 LIs, whereas some patients with low‐grade meningiomas could have higher MIB‐1 LIs (Figure 5). Atypical or malignant meningiomas do not require higher MIB‐1 LIs for diagnosis. The use of MIB‐1 LI as a prognostic indicator in meningioma has been the subject of many studies. Most, but not all, studies have found significant differences between the MIB‐1 labeling indices of benign, atypical, and anaplastic meningiomas. The means, ranges, and cutoff values for the prediction of meningioma recurrence have not been established. From 3 to 10% or more of MIB‐1 LIs of meningiomas have been reported to be related to higher recurrence rates or to be a prognostic factor for radiosurgical outcomes (Kim et al., 2012; Nakaya et al., 2009), whereas other studies have found that MIB‐1 LI is not useful for predicting tumor recurrence (Tyagi et al., 2004; Zhu et al., 2015). This discrepancy could be explained by the heterogeneous nature of these tumors. Nevertheless, it is difficult to say on the basis of our results which class of DTcIs would be associated with which type of tumor tissue. Further pathological studies of each class by biopsy or resection could clarify the relationship.

The patterns of six DTI parameters in each class were characteristic. Class numbers 13 and 14 of 16‐class DTcIs of the WHO grading showed low DWI values; however, low DWI values were also revealed in lower class numbers. Class number 1, which indicates low‐grade meningiomas, had low FA values, as previously reported (Jolapara et al., 2010; Toh et al., 2008). Nevertheless, it is difficult to predict high‐grade meningiomas by FA values alone. For instance, class numbers 13, 15, and 16, which indicate high‐grade meningioma, also had low FA values, whereas class number 14 had the highest FA values. These findings are consistent with previous studies that showed that low FA values did not always indicate low grades (Santelli et al., 2010; Stadnik et al., 2001). Class number 16, which indicates high‐grade meningioma, had lower FA and higher MD and RD values, contrary to the previous report (Toh et al., 2008). With regard to meningioma grade, there are some contradictory studies. Sanverdi et al. (2012) found no correlation between ADC value and grade. Most previous studies investigated a single MRI parameter or analyzed data individually even if they obtained several parameters, and hence the results might be controversial. Our results demonstrated several heterogeneous patterns of six DTI parameters that indicated high‐grade meningiomas.

Class numbers 9 and 10 of 10‐class DTcIs for the MIB‐1 classification had low DWI, S0, AD, RD, and MD values, which was consistent with previous reports that showed a relationship between low ADC values and higher MIB‐1 LIs (Tang et al., 2014). Class number 8 also had low DWI values, whereas the S0, AD, RD, and MD values were prominently high. Higher T2 signal intensities were seen significantly more often in higher MIB‐1 LIs and were also significantly correlated with peritumoral brain edema (Kim et al., 2011). With regard to FA, both high and low values were seen in class numbers 8−10, which indicated higher MIB‐1 LIs. Previous studies of brain tumors reported relationships between high FA values and high cellularity, high WHO grades, and high MIB‐1 LIs (Jolapara et al., 2010; Toh et al., 2008). The factors that affect DTI parameters have proved to be microstructures, namely cellularity, nucleus‐to‐cytoplasm ratio, or vascularity, in brain tumors. Nevertheless, the details have not yet been properly analyzed. With regard to proliferative activity, there are some contradictory studies with a single MRI parameter. Zikou et al. (2016) reported that there were no significant correlations between ADC or FA and MIB‐1 LIs. These studies suggest that differentiating MIB‐1 LIs on the basis of only a single parameter would be difficult. In the present study, we used six DTI‐derived parameters in combination, thereby improving classification performance. In addition, Tang et al. (2014) reported that low‐grade meningiomas with higher MIB‐1 LIs and low ADC values had a high recurrence rate. Although the predictors of meningioma recurrence have been studied from the viewpoint of WHO classification, proliferative indices, molecular assessment, etc., the prediction of recurrence is still a challenge during management of meningioma (Tyagi et al., 2004). Observation of changes in tumor size for years is needed to verify the results for the prediction of meningioma recurrence.

Our method is less effective for differentiating between higher and lower MIB‐1 LIs than it is for predicting WHO grade. Although the classification performance is not high in our analysis, regional visual inspections reveal the potential to detect atypical meningiomas with lower MIB‐1 LIs or benign meningiomas with higher MIB‐1 LIs (Figure 5). MIB‐1 LI was calculated by different counting methods in previous reports (Nakasu et al., 2001; Rezanko et al., 2008). Generally, the most densely staining areas were counted, and with another method, randomly selected areas were counted. In the present study, regions with the most immunostaining (known as “hot spots”) were used for the determination of MIB‐1 LI. However, in previous studies, it was observed that MIB‐1 staining was not uniform in the tumors (Bohra et al., 2016; Iuchi et al., 1999). One of the reasons for lower performance in the classification of MIB‐1 LIs is that we analyzed DTcIs from the whole tumor, whereas MIB‐1 LI was determined only from the hot spot. Further studies with regional comparisons between DTcIs and histological findings would improve classification performance.

With regard to the number of classes for the best classification performance, 10‐class DTcIs were the best for MIB‐1 LI classification, whereas 16‐class DTcIs were the best for WHO classification. The smaller number of classes in MIB‐1 LIs is due to the fact that MIB‐1 LIs have been shown to be correlated with cell proliferative potency and cellularity. Class numbers 9 and 10 of 10‐class DTcIs for MIB‐1 classification had low MD values. Several previous studies have reported that ADC values are negatively correlated with cell proliferative potential and cellularity (Alexiou et al., 2014; Tang et al., 2014). Our clustering method with multiple parameters is still promising, because cellularity is not equivalent to MIB‐1 LI, although the MD values affected the result through the close relation between MIB‐1 LI and proliferative potential or cellularity. Consequently, DTcIs with smaller numbers of classes presumably enabled MIB‐1 LI classification. DTcIs with larger numbers of classes (16 classes) used for WHO classification would reflect the diversity of the histological findings of meningiomas. Cellularity is included in the diagnostic criteria for high‐grade meningiomas; however, low‐grade meningiomas do not always have sparse cellularity. Meningothelial meningiomas have comparatively high cellularity, and fibroblastic meningiomas consist of spindle cells forming interlacing bundles in a collagen‐rich matrix, whereas microcystic meningiomas characterized by intracellular microcystic spaces have a sparse distribution of cells (Louis et al., 2007; Louis et al., 2016). These diverse factors other than cellularity could affect the DTI parameters, and hence the best number of classes for classification might be larger for WHO classification.

The present study has several limitations. First, because we did not compare cellularity in low‐grade and high‐grade meningiomas, we could not definitely state that the differences in ADC were due to differences in cellularity. Second, we did not study other, less common subtypes of WHO grade I meningiomas such as microcystic, secretory, and metaplastic meningiomas. We also did not encounter other WHO grade II meningiomas (chordoid and clear cell meningiomas) or grade III meningiomas (rhabdoid meningiomas) during our study period. These less common meningiomas form specific histological architectural patterns (Louis et al., 2007). Therefore, we speculate that their diffusion anisotropy would be different from that of atypical or anaplastic meningiomas that are “featureless” on microscopic examination. Third, as mentioned above, we did not analyze DTI parameters topologically with histopathological findings. Fourth, the study was limited by its small sample size of 27 patients and lack of long‐term follow‐up. A recent radiomics study with large sample size (Duan et al., 2023) reported the differentiation of meningioma grading, using 2D and 3D features obtained only from contrast enhanced T1‐weighted images. The AUC of 2D and 3D models was 0.717–0.773, which was lower than that of our methods using DTcIs. Park et al. (2019) revealed that the combination of T1 images with ADC and FA maps were useful for differentiating meningioma grades. In addition, gadolinium, which is typically used as contrast agents, can cause side effects, such as hypotension or cardiac arrest (Thomsen, 2003), or cannot be used for patients with renal failure. Thus, the radiomics using only contrast enhanced T1‐weighted images might not be used for definitive preoperative grading. In another recent radiomics study with large sample size (She et al., 2023), using three advanced diffusion models: diffusion kurtosis imaging, mean apparent propagator, and neurite orientation dispersion and density imaging, the benefits obtained from these advanced models were small in grading meningiomas, compared with the DTI. In addition, three models are weakly associated with the Ki‐67 proliferation. Further studies will be needed to evaluate the efficacy of new advanced diffusion models. Compared with the recent studies with large sample size, our method might be useful to differentiate the grade of meningiomas, but the prognostic significance could be emphasized by studies with larger sample sizes and long‐term follow‐up.

## 5 CONCLUSION

This study applied a two‐level clustering approach consisting of SOM followed by the KM++ algorithm for unsupervised clustering of a large number of input vectors with multiple features by DTI. The greatest advantage of this method is that it enables clustering images called DTcIs to be obtained, which can be used for the visual grading of meningiomas and to help differentiate not only between low‐ and high‐grade meningiomas but also between meningiomas with high and low MIB‐1 LIs, without pathological information. Our method could lead to more accurate noninvasive prediction of recurrence and more appropriate treatment of brain tumors.

## CLINICAL TRIAL REGISTRATION

None.

## CONFLICT OF INTEREST STATEMENT

None.

### ETHICS STATEMENT AND PATIENT CONSENT

This study was approved by the Ethics Committee of the Kyoto University Graduate School of Medicine (C570), and written informed consent was obtained from all patients.

### PEER REVIEW

The peer review history for this article is available at https://publons.com/publon/10.1002/brb3.3201.

## Data Availability

The data that support the findings of this study are available from the corresponding author upon reasonable request.
